# The population genomic basis of geographic differentiation in North American common ragweed (*Ambrosia artemisiifolia* L.)

**DOI:** 10.1002/ece3.2143

**Published:** 2016-05-05

**Authors:** Michael D. Martin, Morten Tange Olsen, Jose A. Samaniego, Elizabeth A. Zimmer, M. Thomas P. Gilbert

**Affiliations:** ^1^ Centre for GeoGenetics Natural History Museum of Denmark Faculty of Science University of Copenhagen Øster Voldgade 5‐7 1350 Copenhagen K Denmark; ^2^ Center for Theoretical Evolutionary Genomics University of California Valley Life Sciences Building Berkeley California; ^3^ Department of Natural History University Museum Norwegian University of Science and Technology (NTNU) NO‐7491 Trondheim Norway; ^4^ Department of Botany and Laboratories of Analytical Biology National Museum of Natural History Smithsonian Institution Museum Support Center MRC 534, 4210 Silver Hill Road Suitland Maryland 20746; ^5^ Trace and Environmental DNA Laboratory Department of Environment and Agriculture Curtin University Perth Western Australia 6102 Australia

**Keywords:** *Ambrosia artemisiifolia*, common ragweed, GBS, population genetics, population genomics, ragweed

## Abstract

Common ragweed (*Ambrosia artemisiifolia* L.) is an invasive, wind‐pollinated plant nearly ubiquitous in disturbed sites in its eastern North American native range and present across growing portions of Europe, Africa, Asia, and Australia. Phenotypic divergence between European and native‐range populations has been described as rapid evolution. However, a recent study demonstrated major human‐mediated shifts in ragweed genetic structure before introduction to Europe and suggested that native‐range genetic structure and local adaptation might fully explain accelerated growth and other invasive characteristics of introduced populations. Genomic differentiation that potentially influenced this structure has not yet been investigated, and it remains unclear whether substantial admixture during historical disturbance of the native range contributed to the development of invasiveness in introduced European ragweed populations. To investigate fine‐scale population genetic structure across the species' native range, we characterized diallelic SNP loci via a reduced‐representation genotyping‐by‐sequencing (GBS) approach. We corroborate phylogeographic domains previously discovered using traditional sequencing methods, while demonstrating increased power to resolve weak genetic structure in this highly admixed plant species. By identifying exome polymorphisms underlying genetic differentiation, we suggest that geographic differentiation of this important invasive species has occurred more often within pathways that regulate growth and response to defense and stress, which may be associated with survival in North America's diverse climatic regions.

## Introduction

Ecosystem disturbance by human activity has substantially altered the evolutionary trajectories of many plant species. The evolutionary mechanisms that grant some plants the ability to become invasive arise from complex, synergistic processes. These include rapid adaptation to novel environments (Colautti and Barrett 2013; Vandepitte et al. 2014), morphological and other phenological changes (Dlugosch and Parker 2008a), demographic shifts such as bottlenecks and population expansions (Dlugosch and Parker 2008b), changes in genetic structure (Bossdorf et al. 2005), as well as the mode and tempo of introductions from the native range (Baker 1974; Van Kleunen et al. 2010). Introduced genotypes are drawn from the native‐range populations' pools of standing genetic variation; thus, assessment of native‐range patterns of spatial‐genetic variation can potentially enable the reconstruction of the history of introduction of exotic species to new locales (Lavergne and Molofsky 2007; Prentis et al. 2009). When multiple introductions occur from different locations, formerly disparate genotypes have opportunities to form hybrids that may better exploit niches than parent genotypes (Ellstrand and Schierenbeck 2000; Dlugosch et al. 2015).

The North American native common ragweed (*Ambrosia artemisiifolia*; Asteraceae, Compositae) has experienced data remarkable success in Europe, providing opportunities to study the population genetic differentiation between native and introduced ranges (Hodgins and Rieseberg 2011; Hodgins et al. 2013). Ragweed is an important invasive weed that is costly both in terms of its toll on human health and the financial burden of controlling its spread (Storms et al. 1997). Its extremely allergenic airborne pollen is a major cause of allergic rhinitis during flowering season and is forecast to increase in Europe in the near future (Hamaoui‐Laguel et al. 2015). Although today the species is nearly continuously distributed across eastern North America's disturbed landscapes, sedimentary pollen paleo‐records indicate this is a recent phenomenon (Grimm 2001). Specifically, the species' range and population density have increased substantially since European settlement of North America, with historical census data documenting its close association with human activity via the clearance of forests and expansion of agriculture in its native range (Martin 2011; Hodgins 2014).

By analyzing nuclear microsatellite and chloroplast SNP data from historical herbarium specimens, Martin et al. (2014) described differential success of ragweed populations during the westward expansion of intensive agriculture and a continental‐scale shift in the geography of genetic structure that had occurred since approximately 1940. Modern spatial genetic structure showed weak, but significant differentiation of a formerly expanded genetic cluster in the northeast and another more widespread cluster extending throughout southeastern and midwestern USA regions. This rapid major shift is a curiosity that lacks a satisfying explanation, although Martin et al. (2014) did offer some possibilities. One suggestion was a genetic shift in response to reforestation of eastern USA over the last century. Another was introgression of new successful genotypes from western regions of the USA. A final possibility was that, after a cessation of unidirectional, human‐mediated propagule movement, locally adapted western genotypes recovered from dormant seed banks to outcompete introduced eastern genotypes. Regardless of its underlying explanation, Martin et al. (2014) further suggested that prior to common ragweed's introduction worldwide, alteration of native‐range genetic structure might have enhanced the species' invasive abilities via the formation of novel genotypes. This “hybridization in native range” hypothesis contrasts with the traditional view that ragweed's invasiveness rapidly evolved upon introduction to new locales (i.e., the “rapid evolution” hypothesis; Hodgins et al. 2013). Thus, several key questions regarding the demographic and evolutionary history of this noxious weed remain unanswered.

In this study, we utilize population genomic data in order to assess native‐range genetic differentiation of common ragweed populations in more detail. Through principal components analysis (PCA) and estimation of genetic clustering with genotyping‐by‐sequencing (GBS) data, we explore the genomic basis of geographic differentiation in native‐range common ragweed populations. Our approach thus seeks to evaluate the contemporary population structure inferred by Martin et al. (2014), by estimating the degree of population differentiation in GBS data from a similar sample of individuals collected across eastern USA. Subsequently, we performed an enrichment analysis of gene ontology (GO) terms associated with SNPs highly weighted in principal component space in order to investigate the functional basis for geographic differentiation between populations that developed during the recent shift in spatial genetic structure.

## Materials and Methods

### Sample collection and DNA extraction

*A. artemisiifolia* individuals from 37 locations were sampled in 2009‐2010, predominantly from roadsides and agricultural fields across the eastern portion of the species' native range (Table S1). Leaf tissue samples were immediately desiccated by collection into silica gel. Silica‐dried leaf tissue was pulverized with silicon beads in a Qiagen (Hilden, Germany) TissueLyser^®^ and then extracted using plant‐specified reagents with the AutoGen (Holliston, MA, USA) AutoGenprep 965 Automated DNA Isolation System.

### Reduced‐representation library construction and Illumina sequencing

Genotyping‐by‐sequencing (GBS) library construction was performed by the Institute for Genomic Diversity and Computational Biology Service Unit at Cornell University (Ithaca, NY) according to a published protocol (Elshire et al. 2011). Briefly, a total of 190 *A. artemisiifolia* genomic DNA extracts were digested with the restriction enzyme ApeKI (4.5‐bp recognition site *5′*‐GCWGC*‐3′*). Following ligation of unique, individual‐specific indexing adapters, libraries were pooled along with other samples from a different study and multiplex sequenced on two flowcell lanes (95 samples and one blank per lane) on the Illumina (San Diego, CA, USA) HiSeq2000 platform using 100‐bp Single Read chemistry.

### Genomic SNP calling and genotyping

Sequence reads were sorted by individual using GBSX (Herten et al. 2015), and demultiplexed sequence data were processed with the Universal Network‐Enabled Analysis Kit (UNEAK) pipeline (Lu et al. 2013) within the software TASSEL 3.0 (Bradbury et al. 2007). This pipeline enables variant locus identification and genotyping of GBS data lacking a reference genome via (1) reciprocal mapping of Illumina sequences for *de novo* discovery of likely allele pairs at the same loci, (2) employing network analysis and filtration (Lu et al. 2013), and ultimately (3) using a binomial likelihood ratio method (Glaubitz et al. 2014) for quantitative genotype calling at polymorphic sites. We required a minimum minor allele frequency of 5% to retain a SNP locus in later analysis.

Although the pipeline identified 250,425 putative diallelic loci containing at most one SNP, the number of loci genotyped per individual was highly variable, as was the number of taxa in which particular SNPs were genotyped (Fig. S1). Consequently, we further filtered the data set of genotypes by removing all individuals for which fewer than 10,000 SNP positions had been genotyped, leaving 60 individuals from 23 sampling locations. We then filtered loci by removing all loci genotyped in at least 50% of the remaining individuals. These filtering steps yielded 6337 SNP loci, genotyped in 60 individuals, and with a per‐individual mean read depth of 10.8X (Table S1). This 6337‐SNP data set was used in later enrichment tests.

Because the presence of loci in genetic linkage in a genomic data set can lead to the detection of spurious genetic structure, we used PLINK v1.09 (Purcell et al. 2007) to test for linkage disequilibrium (LD) between all pairs of the 6337 filtered SNPs. Within the entire 60‐individual data set, we found only a very small portion (0.070%) of SNP pairs in strong LD (*r* ^2^ ≥ 0.5). However, a majority of SNPs were in strong LD with at least one of the other 6336 SNPs. Thus PLINK‐facilitated LD pruning removed a large portion of the data, and subsequent analyses of genetic structure were performed on a multiple sequence alignment of 2829 SNP sites assessed in 60 individuals spanning eastern North America.

### Genetic structure from genomic data

Genetic structure in the LD‐pruned genomic SNP data set was initially investigated through principal component analysis (PCA) using SMARTPCA within the software package EIGENSOFT v4.2 (Patterson et al. 2006; Price et al. 2006). Only the first two principal components were considered, because their eigenvalues showed a sharp break from the lesser principal components in a scree plot (Fig. S2). In addition, we performed PCA using the 6337‐SNP data set without LD pruning (Fig. S3), identifying the top‐weight SNP that were used in later tests of functional enrichment.

ADMIXTURE v1.22 (Alexander et al. 2009) was used on the LD‐pruned data to identify genetic structuring through simultaneous estimation of population allele frequencies and ancestry proportions. A total of 20 randomly seeded replicate runs with fivefold cross‐validation (CV) were each performed using a different assumed number of clusters *K* from 1 to 15. Normally, the value of *K* with the lowest CV error should be chosen as the best fit to the data. For our data, however, CV error was uninformative as it increased with the number of clusters. Thus, we applied the STRUCTURE HARVESTER (Earl and vonHoldt 2012) implementation of the Evanno et al. (2005) method to choose the value of *K* that shows a modal peak in the absolute value of the second‐order rate of change of L(*K*). For each value of *K*, the replicate of highest likelihood was chosen for further analysis as well as plotting.

The R packages adegenet v2.0.1 (Jombart 2008; Jombart and Ahmed 2011) and hierfstat v0.4.18 (Goudet 2005) were used to calculate pairwise differentiation (as *F*_ST_; Nei 1987) between identified population clusters at each genomic locus, and significance (*P *<* *0.01) was assessed via permutation tests (*n *=* *99) in which individual cluster assignments were randomly permuted. The R package pegas v0.8.1 (Paradis 2010) was used to perform exact tests of Hardy–Weinberg equilibrium (HWE) using 1000 Markov chain–Monte Carlo replicates. Rejection of HWE was established at the *P *<* *0.01 level of significance.

### Association of genomic SNPs with transcriptome sequences

Given the geographic signal in our analyses of genetic structure, we thought that SNP loci making large contributions to primary principal components may fall within genes that are geographically divergent in ragweed populations. In order to further explore the genomic basis of geographic differentiation in common ragweed, we tested whether specific top‐weight SNPs were carried by transcripts enriched for genes associated with particular molecular functions. Specifically, individual SNP loci were ranked by the absolute value of their loading/weight in the first two principal component vectors from the 6337‐SNP PCA, and the 5% highest‐weight SNPs were retained for analysis. The 33‐ to 64‐bp nucleotide sequences (hereafter “tags”) flanking sequenced ApeKI restriction sites associated with these SNPs were used in a BLASTALL v2.2.26 (Zhang et al. 2000) blastn search against an un‐annotated, normalized, 33.2‐Mbp expressed sequence tag (EST) library assembly derived from a single North American *A. artemisiifolia* individual collected in Minnesota (Lai et al. 2012), using an E‐value threshold of 10^−10^ and reporting at most five best hits. Transcript sequences that matched the entire length of restriction site‐associated tag sequences with greater than 95% identity were chosen for further analysis. In some cases, the entire length of a query tag sequence did not align to a target transcript. These transcripts were included if there was greater than 95% identity between the two sequences when all unaligned bases were considered mismatches. In cases where tag sequences matched equally well to multiple transcripts, all transcript matches were included in the enrichment analysis. This process resulted in a base set of 1921 tag alignments to the transcriptome assembly, which was used to construct a base reference set for each of the enrichment tests described below.

### Tests of functional enrichment from genetic structure

Using blastn, nucleotide sequences of transcript hits were used as queries in a blastx search using against the NCBI nonredundant (nr) protein database with an E‐value threshold of 10^−10^ and limited to 20 hits per query sequence. The software BLAST2GO pipe v2.5.0 (Conesa et al. 2005; Conesa and Götz 2008) was used to associate GO terms with each hit. In the BLAST2GO analysis, we used an annotation rule cutoff of 55 and a GO weight of five. BLAST results were imported into BLAST2GO Pro (v. 3.1.3), and sequence GO term annotations were processed using Annex annotation augmentation, ensuring that each branch's lowest term alone remained in the set of annotations. The transcriptome assembly published by Lai et al. (2012) consists of 62,946 sequences. A total of 38,307 of these could be annotated with GO terms in the absence of an annotated ragweed transcriptome, so further analysis of transcriptome assembly was limited to the annotated sequences. Fisher's exact tests were used to identify enrichment in GO terms associated with transcripts to which various test sets of outlier SNPs (identified in analyses of genetic structure based on both PCA and ADMIXTURE) were well aligned. In each test, a test‐specific reference set was constructed by excluding genes common to the base reference set (described above) and the test set, and a multiple‐measure false discovery rate (FDR) filter was applied with a significant cutoff of *P *<* *0.05.

## Results

### Illumina sequencing, SNP discovery, and genotyping

Two lanes of Illumina sequencing of the 190 samples produced 381.5M sequence reads passing Illumina filters. Of these, 298.4M contained an unambiguous, individual‐specific barcode. The number of sequences per pooled individual was highly variable (Fig. S4), which is consistent with both nonequimolar sample pooling and unequal library performance in the sequencing run. We note that *Ambrosia* species are known for an abundance of diversity of terpenoid compounds in their tissues (Herz et al. 1969; Martino et al. 2015), so the differential success of samples may have resulted from coextraction of varying amounts of these residual compounds that can interfere with downstream enzyme reactions.

Sequence data processing with the UNEAK pipeline revealed that 205.5M (53.8% of total) reads carried both the ApeKI restriction site and an unambiguous barcode. The number of unique diallelic, single‐polymorphism tags identified by TASSEL's network analysis was 4,824,895, which is approximately equal to twice the expected number of ApeKI cut sites in the ~1.1‐Gbp *A. artemisiifolia* genome (1.1 Gbp/(4^4.5^) = 2.15M; Kubešová et al. 2010). Each of these tags was observed 32.9 ± 1113 (mean ± SD) times, indicating that mean sequencing depth in this data set was low and highly variable. We excluded insertion/deletion‐based tags, considering only diallelic, single‐SNP tags in further analyses. Thus, after filtering, the dataset consisted of 6337 SNP loci, genotyped in 60 individuals.

### Population genetic structure

Consistent with previous observations based on nuclear microsatellite loci (e.g., Genton et al. 2005; Gladieux et al. 2010; Chun et al. 2011; Martin et al. 2014), we observed widespread heterozygote deficiency in the discovered nuclear loci. Considering the 60 individuals as a single meta‐population, HWE was rejected for 46.6% of the genomic loci (Fig. S5). Deviations from HWE are commonly observed in *A. artemisiifolia* populations and have been discussed extensively (e.g., Martin et al. 2014; Genton et al. 2005).

**Figure 1:**
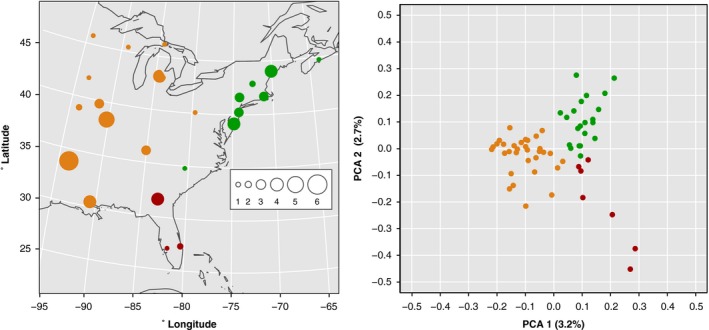
Multivariate analysis of genomic SNP data segregates samples by geographic sampling location. Left, the number of study individuals sampled from each population is indicated by the size of the circle. Right, the first two principal coordinate axes differentiate individual samples by their source location from western (orange), southeastern (red), or northeastern (green) genetic clusters.

To reduce the dimensionality of our genomic SNP data set and to obtain initial view of genetic structuring in our data, the 2829 filtered, LD‐pruned SNP variants from 60 individuals were used to perform a PCA. This analysis revealed that the first two principal component axes reflect the geography of wild populations remarkably well, while accounting for around 6% of the samples' genomic variance (Fig. 1). Individual sample positions on the principal coordinate axes clustered in a pattern that corresponds to their geographic sampling and that could be subjectively divided into three clusters of individuals uniting populations from: Florida and southern Georgia (southeastern); along the Atlantic Coast from Tennessee to New Brunswick (northeastern); all the remaining area (western). Differentiation of the western and northeastern clusters (*F*_ST_ = 0.010, *P *<* *0.01) was low and half that of the western and southeastern clusters (*F*_ST_ = 0.012, *P *<* *0.01; Table S2).

**Figure 2:**
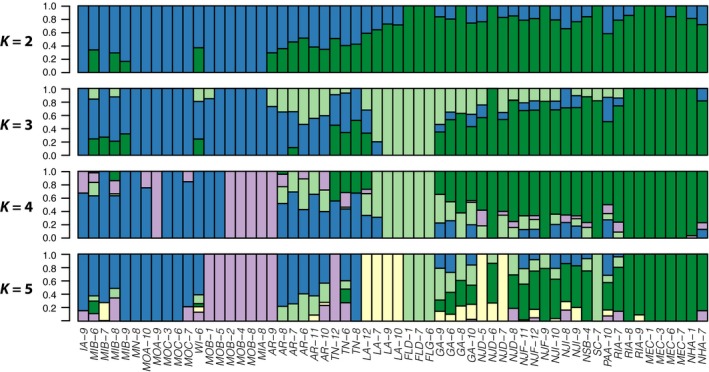
Portion of each ragweed individual's genome assigned to a number of ancestral clusters from *K *=* *2 to *K *=* *5.

**Figure 3:**
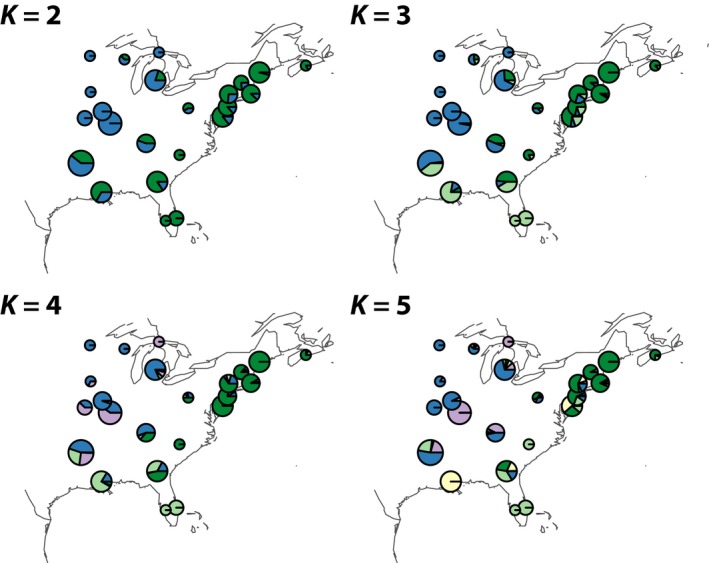
Ancestral genetic cluster assignment frequency in different locations for *K *=* *2 to *K *=* *5. The area of each pie scales linearly with the number of individuals sampled from that location. Pie colors are consistent with cluster assignments of Figure 2.

Estimation of ancestry proportions with ADMIXTURE showed that a single, panmictic genetic cluster is the most likely, although lower cross‐validation errors were achieved with very high values of *K* approaching the number of individuals in the data set (Fig. S6). Implementation of the Evanno et al. (2005) method revealed a clear peak in ∆K when *K *=* *2, suggesting the most likely scenario involves recent admixture of two ancestral genetic clusters (Fig. S7), although the assigned proportions of genetic ancestry were strongly correlated with geography for *K *=* *2–5 (Figs. 2, 3). When *K *=* *2, individuals segregate into weakly differentiated eastern and western clusters (*F*_ST_ = 0.0109, *P *<* *0.01) whose diffuse boundary is approximately aligned along the axis of the Appalachian Mountains. The ancestries of individuals from Arkansas, Louisiana, and Tennessee indicate roughly 50% admixture.

When *K *=* *3, the eastern cluster subdivides into northeastern and southeastern clusters, and the three genetic clusters are more highly differentiated (pairwise *F*_ST_ = 0.012–0.019) than the *K *=* *2 clusters (Table S3). The southeastern cluster is the most divergent and encompasses individuals from Florida, Louisiana, Georgia, and Arkansas, with mixed ancestry appearing in all populations except those in Florida. When *K *=* *4, the western genetic cluster subdivides into southwestern and midwestern clusters that are mixed throughout the western range and more poorly correlated with geography. These four clusters are the most highly differentiated (mean pairwise *F*_ST_ = 0.022, *P *<* *0.01), with the midwestern cluster most divergent (Table S4). At *K *=* *5, individuals collected in Louisiana are assigned their own ancestral cluster, and at *K *>* *5, geographic correlations appear to break down.

### Functional basis of population genomic and geographic differentiation

The 5% top‐weight loci from the principal components analysis were represented by 632 tag sequences, 38–64 bp in length. The BLAST analysis aligning these sequences with the native transcriptome assembly identified 222 transcript sequences with unambiguous homology to the query tag sequences. Transcripts from this data set were also enriched for particular GO terms of varied function, including defense response, biosynthesis and metabolism of triterpenoids, catabolysis of glucosinolates (aromatic compounds related to defense against pathogens and herbivores), regulation of growth, RNA metabolism, sulfur/phosphorus metabolism, response to the plant growth hormone indolebutyric acid, processes relating to protein localization to the mitochondrion, and drug transport/responses (Table 1).

**Table 1:** Gene ontology (GO) terms identified by enrichment analysis of annotated transcripts harboring top‐weight SNPs from analysis of principal components in North American ragweed genetic data

GO‐ID	Term	Category	FDR	*P*‐value	*N*_T_	*N*_R_	Over/Under
GO:0006626	Protein targeting to mitochondrion	P	6.09E‐03	5.04E‐06	6	10	OVER
GO:0072655	Establishment of protein localization to mitochondrion	P	6.09E‐03	5.04E‐06	6	10	OVER
GO:0070585	Protein localization to mitochondrion	P	6.09E‐03	5.04E‐06	6	10	OVER
GO:0016145	S‐glycoside catabolic process	P	6.09E‐03	1.28E‐05	4	2	OVER
GO:0042343	Indole glucosinolate metabolic process	P	6.09E‐03	1.28E‐05	4	2	OVER
GO:0042344	Indole glucosinolate catabolic process	P	6.09E‐03	1.28E‐05	4	2	OVER
GO:0019759	Glycosinolate catabolic process	P	6.09E‐03	1.28E‐05	4	2	OVER
GO:0019762	Glucosinolate catabolic process	P	6.09E‐03	1.28E‐05	4	2	OVER
GO:0043407	Negative regulation of MAP kinase activity	P	6.09E‐03	1.28E‐05	4	2	OVER
GO:0043409	Negative regulation of MAPK cascade	P	6.09E‐03	1.28E‐05	4	2	OVER
GO:0071366	Cellular response to indolebutyric acid stimulus	P	6.09E‐03	1.28E‐05	4	2	OVER
GO:0052545	Callose localization	P	6.09E‐03	1.28E‐05	7	20	OVER
GO:0033037	Polysaccharide localization	P	7.13E‐03	1.66E‐05	7	21	OVER
GO:0052542	Defense response by callose deposition	P	7.13E‐03	2.20E‐05	6	14	OVER
GO:0006722	Triterpenoid metabolic process	P	7.13E‐03	2.84E‐05	5	8	OVER
GO:0016104	Triterpenoid biosynthetic process	P	7.13E‐03	2.84E‐05	5	8	OVER
GO:0019742	Pentacyclic triterpenoid metabolic process	P	7.13E‐03	2.84E‐05	5	8	OVER
GO:0019745	Pentacyclic triterpenoid biosynthetic process	P	7.13E‐03	2.84E‐05	5	8	OVER
GO:0071900	Regulation of protein serine/threonine kinase activity	P	7.13E‐03	2.84E‐05	5	8	OVER
GO:0051348	Negative regulation of transferase activity	P	7.13E‐03	2.91E‐05	4	3	OVER
GO:0006469	Negative regulation of protein kinase activity	P	7.13E‐03	2.91E‐05	4	3	OVER
GO:0033673	Negative regulation of kinase activity	P	7.13E‐03	2.91E‐05	4	3	OVER
GO:0071901	Negative regulation of protein serine/threonine kinase activity	P	7.13E‐03	2.91E‐05	4	3	OVER
GO:0007005	Mitochondrion organization	P	7.13E‐03	3.00E‐05	6	15	OVER
GO:0042326	Negative regulation of phosphorylation	P	1.12E‐02	5.67E‐05	4	4	OVER
GO:0043405	Regulation of MAP kinase activity	P	1.12E‐02	5.67E‐05	4	4	OVER
GO:0010563	Negative regulation of phosphorus metabolic process	P	1.12E‐02	5.67E‐05	4	4	OVER
GO:0045936	Negative regulation of phosphate metabolic process	P	1.12E‐02	5.67E‐05	4	4	OVER
GO:0001933	Negative regulation of protein phosphorylation	P	1.12E‐02	5.67E‐05	4	4	OVER
GO:0000469	Cleavage involved in rRNA processing	P	1.78E‐02	9.97E‐05	4	5	OVER
GO:0000478	Endonucleolytic cleavage involved in rRNA processing	P	1.78E‐02	9.97E‐05	4	5	OVER
GO:0090502	RNA phosphodiester bond hydrolysis, endonucleolytic	P	1.78E‐02	9.97E‐05	4	5	OVER
GO:0006855	Drug transmembrane transport	P	1.82E‐02	1.12E‐04	6	20	OVER
GO:0042493	Response to drug	P	1.82E‐02	1.12E‐04	6	20	OVER
GO:0015893	Drug transport	P	1.82E‐02	1.12E‐04	6	20	OVER
GO:0031400	Negative regulation of protein modification process	P	2.37E‐02	1.62E‐04	4	6	OVER
GO:0071417	Cellular response to organonitrogen compound	P	2.37E‐02	1.62E‐04	4	6	OVER
GO:1901658	Glycosyl compound catabolic process	P	2.37E‐02	1.62E‐04	4	6	OVER
GO:0015086	Cadmium ion transmembrane transporter activity	F	2.37E‐02	1.62E‐04	4	6	OVER
GO:0052543	Callose deposition in cell wall	P	3.08E‐02	2.21E‐04	5	14	OVER
GO:0052386	Cell wall thickening	P	3.08E‐02	2.21E‐04	5	14	OVER
GO:1902532	Negative regulation of intracellular signal transduction	P	3.30E‐02	2.49E‐04	4	7	OVER
GO:0080026	Response to indolebutyric acid	P	3.30E‐02	2.49E‐04	4	7	OVER
GO:0006839	Mitochondrial transport	P	3.38E‐02	2.60E‐04	6	24	OVER
GO:0042299	Lupeol synthase activity	F	3.48E‐02	2.80E‐04	3	2	OVER
GO:0031559	Oxidosqualene cyclase activity	F	3.48E‐02	2.80E‐04	3	2	OVER
GO:0051248	Negative regulation of protein metabolic process	P	3.99E‐02	3.64E‐04	4	8	OVER
GO:0052544	Defense response by callose deposition in cell wall	P	3.99E‐02	3.64E‐04	4	8	OVER
GO:0015691	Cadmium ion transport	P	3.99E‐02	3.64E‐04	4	8	OVER
GO:0052482	Defense response by cell wall thickening	P	3.99E‐02	3.64E‐04	4	8	OVER
GO:0032269	Negative regulation of cellular protein metabolic process	P	3.99E‐02	3.64E‐04	4	8	OVER
GO:0044273	Sulfur compound catabolic process	P	3.99E‐02	3.64E‐04	4	8	OVER
GO:0002831	Regulation of response to biotic stimulus	P	4.84E‐02	4.49E‐04	6	27	OVER

Single‐test *P*‐values are shown. *N*_T_, number in test group; *N*_R_, number in reference group; P, biological process; F, molecular function.

## Discussion

It has been suggested that populations of *A. artemisiifolia* experienced rapid evolution upon introduction to Europe, increasing its success as an invasive after multiple introductions from different source populations (Chun et al. 2011; Hodgins and Rieseberg 2011; Hodgins et al. 2013). However, based on genetic clustering observed within the species' present‐day eastern North American range, Martin et al. (2014) suggested that the signal of rapid adaptation reported by Hodgins et al. (2013) might be explained simply by incomplete sampling of the structured genetic diversity present in North America. For proper comparisons of native and introduced populations, assessing the extent of population genetic differentiation in common ragweed's native range is important for understanding the evolutionary processes that were at play during the plant's introductions to Europe.

Complicating our understanding, we know that human‐mediated shifts in *A. artemisiifolia* genetic structure occurred very recently (Martin et al. 2014). Novel genotypic combinations produced by mating between diverse, divergent populations may produce populations able to arrive rapidly at new fitness optima. By characterizing the correlation of geography and native‐range population genetic differentiation, we can begin to understand how genotypes introduced from different sources may fare in novel locales.

### Geographic differentiation of ragweed populations

Not surprisingly, we have shown that our genomic SNP data have increased power to detect population genetic structure over a small number of traditional microsatellites and chloroplast SNPs used in previous studies (Genton et al. 2005; Gaudeul et al. 2011; Martin et al. 2014). In this report, we used PCA and population genetic clustering‐based analyses to identify weak genetic structure with clear geographic components. The degree of genetic structure we identify is on the order of previous estimates (Genton et al. 2005; Gaudeul et al. 2011; Martin et al. 2014). Our major findings are that at least two major genetic clusters exist in the native range, and further genetic differentiation makes it possible to identify up to five clusters.

This finer‐scale genetic differentiation in North American *A. artemisiifolia* populations at regional scales was supported by both PCA and allele frequency‐based clustering analyses using ADMIXTURE. Geographic mirroring of the principal genetic components space has been observed in genomic data, most notably in SNP data from human populations in Europe (Novembre et al. 2008; Leslie et al. 2015). However, this clear geographic signal was somewhat unexpected for a wind‐pollinated, pioneer plant with high potential for dispersal, and especially in *A. artemisiifolia*, which has also experienced extreme migration and population admixture in the recent past.

The geographic division between the genetic clusters defined here fits well the pattern of the Appalachian Mountain discontinuity described by Soltis et al. (2006), which reviewed a number of species whose populations were shown to have east–west phylogeographic breaks defined by the Appalachian Mountain range and the Apalachicola/Chattahoochee River drainages. Indeed, river systems are a major dispersal mechanism for ragweed and thus would be likely to reinforce phylogeographic break points (Fumanal et al. 2007; Lavoie et al. 2007). This overall phylogeographic pattern also has been seen in other plant species, including Virginia pine (*Pinus virginiana*), Atlantic white cedar (*Chamaecyparis thyoides*; Mylecraine et al. 2004), and the American groundnut (*Apios americana*; Joly and Bruneau 2004). Although these patterns were previously explained in the context of expansions out of glacial refugia, we suspect similar genomic patterns of differentiation could develop between populations due to differential environmental stresses, as discussed below.

### Investigating the functional basis of geographic differentiation

The low density of genomic markers utilized in our study, as well as our inclusion of SNPs in potential genetic linkage, introduces some caveats for interpreting the enriched functional pathways we identify. Pruning or “thinning” loci in LD is a common way to avoid the detection of spurious genetic structure in data sets that contain linked loci. However, this conservative approach is wasteful in that it discards a large fraction of potentially informative sites (Zou et al. 2010) and could be highly deleterious for inference of functional enrichment in our low‐depth GBS study of only filtered 6337 SNPs. This is especially true in the absence of a reference genome, as it is not possible to test directly whether SNP loci are physically clustered along the chromosome and additional stringency in LD pruning is necessary. Perhaps partially due to recent admixture of *A. artemisiifolia* populations, a substantial portion of this study's SNP markers depart from HWE, and statistical independence of alleles cannot be assumed at loci being tested for LD. But because measures of LD are poorly understood under HWE departure in unphased data, unfortunately common LD tests are not necessarily appropriate for this data set (Schaid 2004).

Our tests detected strong LD in only 0.070% of all SNP pairs, and we found that the substantial correlation of genetic structure with geography is robust to the removal of linked SNPs. Under these considerations, we hypothesized that pruning informative markers could be more detrimental to our study of functional enrichment than the inclusion of several SNPs potentially in genetic linkage. Thus, we elected to perform the functional enrichment analyses using the unpruned data set, and our methods may identify particular SNPs underlying geographic differentiation when selection is actually acting on loci with which they are in genetic linkage. To mitigate this confounding issue, our functional enrichment tests were performed not with all annotated *A. artemisiifolia* transcripts, but instead with a reference subset of those associated with our 6337 filtered GBS tags.

General stress response (light stress and oxidative stress) and biosynthesis of plant secondary compounds (defense against pathogens and herbivores) were previously identified as major factors in gene expression differences between introduced ragweed populations and native populations sampled from southeastern Canada and midwestern USA (Hodgins et al. 2013). In agreement, our analysis of SNPs associated with extreme geographic genetic variation identified transcripts involved in diverse processes, especially metabolism of glucosinolates and terpenoids, defense response, and regulation of growth and development, implying that genetic differentiation in genes related to these pathways partly underlie geographic variation between *A. artemisiifolia* populations in their native range. Overrepresentation of GO terms relating to processes within the mitochondrion indicates that differentiation in basic energetics also may be at play.

As Hodgins et al. (2013) found enrichment of RNA metabolism‐associated transcripts across all of their light‐ and nutrient‐stress treatments, our observations of significant over‐representation of transcripts involving RNA processing and metabolism were not surprising. Major fluctuations in RNA stability are known to occur in plant regulatory responses to stress (Narsai et al. 2007). It may be that environmental stressors drive adaptation to the diverse habitats and seasonal extremes encompassed by this species' eastern North American range. These extremes are particularly pronounced in the Great Plains region, which, with its shorter growing season and higher variance in precipitation and temperature, likely presents a more challenging environment for common ragweed (Grimm 2001; Hodgins et al. 2013). We suspect that, in addition phylogeographic patterns formed by mountains and river drainages, adaption to local environments is a major driver of the genetic differentiation among geographically distinct *A. artemisiifolia* populations. The results from Martin et al. (2014) suggest that the current genetic structure has developed just in the past ~100 years, following an extensive westward expansion of a historical northeastern genetic cluster associated with the peak of deforestation in the US around the year 1900. This rapid change is interesting and calls for future work with genomic data from herbarium collections. Those samples may reveal further population genetic structure in past populations, which could further elucidate the role of human‐mediated admixture in the history of this successful invasive species.

### Final remarks

Here, we provide a preliminary catalog of SNP loci involved with local differentiation in ragweed's native range. These SNPs should be targets for further investigation in larger population samples from both North America and introduced ranges around the world. However, in this study, we analyzed only approximately 6300 SNPs, many of which do not fall within coding regions. Much more information could be gleaned from the entire catalog of transcriptome SNPs. An expanded approach that sequences more genomic loci from larger population samples would likely yield very interesting results with regard to the functional basis of the differential success of these populations.

Our study provides clues about the basis for the large phylogeographic shift observed in North American common ragweed populations over the last 80 years (Martin et al. 2014). Our enrichment analyses of GO terms suggest that environmental selection (local adaptation; Savolainen et al. 2013) on genes within pathways involved in responses to stress and defense factors may have been a major determinant of geographic genetic variation in present‐day populations. These important factors might be both biotic and abiotic, including local differences in herbivory, microbial pathogenesis, and the considerable diversity of eastern North American climate regimes. However, the enriched GO terms we report will not always indicate adaptation to local conditions because of the potential effects of genetic linkage, population differentiation is often nonadaptive, and local adaptation in the native range has not been established in ragweed. As a result, the evolutionary processes and genomic loci we identify should be investigated with whole‐genome sequencing or genomic data sets of higher density and sequencing depth.

Still we lack a definitive explanation for the curious historical distribution of a human‐associated ragweed genetic cluster that apparently expanded from the northeastern USA. Other researchers should seek to obtain genomic data from historical collections of this species in order to identify high‐resolution temporal trends in population genetic structure. Future efforts should characterize loci that may have been under differential selection in the past, especially regarding the human‐associated cluster that may have gone on to invade Western Europe.

## Data Accessibility

Sequence data generated for this study have been deposited at the European Nucleotide Archive under accession code PRJEB13190.

## Conflict of Interest

None declared.

## Supporting information


**Figure S1.** The number of genotyped loci per sample after analysis of sequencing data with the UNEAK pipeline.
**Figure S2.** Eigenvalues for each principal component axis of the filtered genomic SNP genotype alignment dataset.
**Figure S3.** Multivariate analysis of genomic 6337 SNPs segregates samples by geographic sampling location.
**Figure S4.** Sample‐specific bias in the number of generated sequence reads with expected barcodes.
**Figure S5.** Distribution of exact tests for Hardy‐Weinberg Equilibrium at all 6337 genomic loci using 1000 MCMC replicates.
**Figure S6.** Mean cross‐validation (CV) error for different numbers of assumed ancestral population clusters (*K*).
**Figure S7.** Implementing the Evanno et al. (2005) method for determining the optimal value of *K* genetic clusters.
**Table S1.** Provenance of *A. artemisiifolia* individuals included in this study.
**Table S2.** Pairwise *F*_ST_ estimated between three genetic clusters defined by principal components analysis.
**Table S3.** Pairwise *F*_ST_ estimated between genetic clusters defined by ADMIXTURE analysis with *K *=* *3.
**Table S4.** Pairwise *F*_ST_ estimated between genetic clusters defined by ADMIXTURE analysis with *K *=* *4.
