# Supplementary material for: The population genomic basis of geographic differentiation in North American common ragweed (Ambrosia artemisiifolia L.)
Source: Ecol Evol. 2016 May 5;6(11):3760–71. doi: 10.1002/ece3.2143 (PMC5513308; doi:10.1002/ece3.2143)
Supplement: Supplementary file 1 — Figure S1. The number of genotyped loci per sample after analysis of sequencing data with the UNEAK pipeline. Figure S2. Eigenvalues for each principal component axis of the filtered genomic SNP genotype alignment dataset. Figure S3. Multivariate analysis of genomic 6337 SNPs segregates samples by geographic sampling location. Figure S4. Sample‐specific bias in the number of generated sequence reads with expected barcodes. Figure S5. Distribution of exact tests for Hardy‐Weinberg Equilibrium at all 6337 genomic loci using 1000 MCMC replicates. Figure S6. Mean cross‐validation (CV) error for different numbers of assumed ancestral population clusters (K). Figure S7. Implementing the Evanno et al. (2005) method for determining the optimal value of K genetic clusters. Table S1. Provenance of A. artemisiifolia individuals included in this study. Table S2. Pairwise F ST estimated between three genetic clusters defined by principal components analysis. Table S3. Pairwise F ST estimated between genetic clusters defined by ADMIXTURE analysis with K = 3. Table S4. Pairwise F ST estimated between genetic clusters defined by ADMIXTURE analysis with K = 4. [file ECE3-6-3760-s001.docx]

*Supplementary figures and tables*


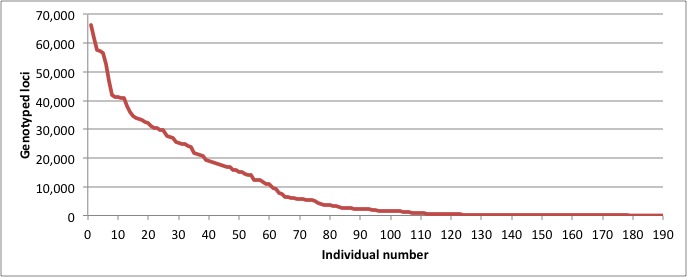


**Supplementary Fig. 1**. The number of genotyped loci per sample after analysis of sequencing data with the UNEAK pipeline.

**Supplementary Fig. 2**. Eigenvalues for each principal component axis of the filtered genomic SNP genotype alignment dataset.

**Supplementary Fig. 3**. Multivariate analysis of genomic 6,337 SNPs segregates samples by geographic sampling location. **Left**, the number of study individuals sampled from each population is indicated by the size of the circle. **Right**, the first two principal coordinate axes differentiate individual samples by their source location from Western (orange), Southeastern (red), or Northeastern (green) genetic clusters.

**Supplementary Fig. 4**. Sample-specific bias in the number of generated sequence reads with expected barcodes.

**Supplementary Fig. 5**. Distribution of exact tests for Hardy-Weinberg Equilibrium at all 6,337 genomic loci using 1000 MCMC replicates. All sampling locations were pooled for the tests.

**Supplementary Fig. 6**. Mean cross-validation (CV) error for different numbers of assumed ancestral population clusters (*K*). Vertical bars indicate ±1 standard deviation of CV error from 20 replicates of ADMIXTURE analysis.

**Supplementary Fig. 7**. Implementing the Evanno *et al*. (2005) method for determining the optimal value of *K* genetic clusters. **a**, Log-likelihood of 20 replicate runs represented by a boxplot. **b**, First-order rate of change of log-likelihood. **c**, Second-order rate of change of log-likelihood. **d**, ∆K.

**Supplementary** **Table 1**. Provenance of *A. artemisiifolia* individuals included in this study. Samples below the grey bar were not included in the 60-individual filtered dataset analyzed in this study.

| **Sampling location** | **(Latitude, longitude)** | **Sample ID** | **Mean depth of 6,337-SNP panel (X)** | **Flowcell** | **Barcode** |
| --- | --- | --- | --- | --- | --- |
| Ozan, Arkansas | (33.841883,  -93.77575) | AR-10 | 13.7 | D0U2VACXX | CCATGGGT |
|  |  | AR-11 | 10.6 | D0U2VACXX | CGCGGAGA |
|  |  | AR-6 | 25.4 | D0U2VACXX | TGCTGGA |
|  |  | AR-7 | 6.5 | D0U2VACXX | AACCGAGA |
|  |  | AR-8 | 16.5 | D0U2VACXX | ACAGGGAA |
|  |  | AR-9 | 4.6 | D0U2VACXX | ACGTGGTA |
| Fort Pierce, Florida | (27.374365,  -80.416042) | FLD-1 | 18.1 | C1B5JACXX | CGAT |
|  |  | FLD-7 | 20.5 | C1B5JACXX | GCTGTGGA |
| Arcadia, Florida | (27.085217,  -81.794967) | FLG-6 | 5.2 | D0U2VACXX | ATATGT |
| Fitzgerald, Georgia | (31.678667,  -83.196133) | GA-10 | 16.7 | D0U2VACXX | TGCAAGGA |
|  |  | GA-6 | 25.0 | D0U2VACXX | GGTGT |
|  |  | GA-8 | 12.5 | D0U2VACXX | AGTGGA |
|  |  | GA-9 | 17.9 | D0U2VACXX | TAGGCCAT |
| Cambridge, Iowa | (41.874983, -93.482783) | IA-9 | 7.0 | D0U2VACXX | GAGGA |
| Holden, Louisiana | (30.468783,  -90.6429) | LA-10 | 3.5 | D0U2VACXX | TAGCGGA |
|  |  | LA-12 | 9.7 | D0U2VACXX | TCTGTGA |
|  |  | LA-7 | 8.3 | D0U2VACXX | CGGTAGA |
|  |  | LA-9 | 3.7 | D0U2VACXX | GCGGAAT |
| Lisbon, Maine | (44.013321,  -70.069435) | MEC-1 | 4.3 | C1B5JACXX | ACTA |
|  |  | MEC-3 | 25.9 | C1B5JACXX | GAGATA |
|  |  | MEC-6 | 9.9 | C1B5JACXX | ATATGT |
|  |  | MEC-7 | 6.1 | C1B5JACXX | ATTAATT |
| Pickford, Michigan | (46.172383,  -84.36515) | MIA-8 | 3.9 | D0U2VACXX | ATGCCT |
| Ashley, Michigan | (43.153767,  -84.503683) | MIB-6 | 18.4 | D0U2VACXX | TGGCTA |
|  |  | MIB-7 | 13.3 | D0U2VACXX | ACGTGTT |
|  |  | MIB-8 | 10.1 | D0U2VACXX | ATTAATT |
|  |  | MIB-9 | 9.3 | D0U2VACXX | ATTGGAT |
| Rice, Minnesota | (45.793567,  -94.24465) | MN-8 | 2.5 | D0U2VACXX | GATC |
| Odessa, Missouri | (38.968433,  -93.879417) | MOA-10 | 3.6 | D0U2VACXX | TATCGGGA |
|  |  | MOA-9 | 2.4 | D0U2VACXX | GTGAGGGT |
| St. Louis, Missouri | (38.386347, -90.25932) | MOB-1 | 1.1 | C1B5JACXX | TGCGA |
|  |  | MOB-2 | 2.6 | C1B5JACXX | GGTTGT |
|  |  | MOB-4 | 5.6 | C1B5JACXX | TAGGCCAT |
|  |  | MOB-5 | 1.3 | C1B5JACXX | CCTAC |
|  |  | MOB-8 | 4.9 | C1B5JACXX | CGTGTGGT |
| Hannibal, Missouri | (39.709343,  -91.500199) | MOC-3 | 2.0 | C1B5JACXX | CTTGCTT |
|  |  | MOC-6 | 1.8 | C1B5JACXX | CGCGGT |
|  |  | MOC-7 | 5.6 | C1B5JACXX | CGCTGAT |
| Chesterfield, New Hampshire | (42.890685,  -72.527665) | NHA-1 | 12.3 | C1B5JACXX | ACAAA |
|  |  | NHA-7 | 9.0 | C1B5JACXX | TCTGTGA |
| Middle Township, New Jersey | (39.149171,  -74.851037) | NJD-5 | 4.3 | C1B5JACXX | AGGC |
|  |  | NJD-6 | 6.4 | C1B5JACXX | TCGTT |
|  |  | NJD-7 | 6.4 | C1B5JACXX | TGGCTA |
|  |  | NJD-8 | 3.7 | C1B5JACXX | TGCTGGA |
| Freehold, New Jersey | (40.226333,  -74.257833) | NJF-11 | 23.5 | D0U2VACXX | AACT |
|  |  | NJF-12 | 24.1 | D0U2VACXX | GCGT |
|  |  | NJF-9 | 3.9 | D0U2VACXX | ACTA |
| Wallkill, New York | (41.629,  -74.18235) | NJI-10 | 25.7 | D0U2VACXX | ACCGT |
|  |  | NJI-8 | 10.2 | D0U2VACXX | GTATT |
|  |  | NJI-9 | 14.0 | D0U2VACXX | CTGTA |
| Lower Sackville, Nova Scotia | (44.754723,  -63.671654) | NSB-4 | 15.7 | C1B5JACXX | TCTCAGTC |
| Connellsville, Pennsylvania | (40.0785,  -79.61485) | PAA-10 | 20.6 | D0U2VACXX | CTTCCA |
| Fall River, Massachusetts | (41.683117,  -71.134283) | RIA-7 | 20.8 | D0U2VACXX | GGTTGT |
|  |  | RIA-8 | 10.1 | D0U2VACXX | CCAGCT |
|  |  | RIA-9 | 37.7 | D0U2VACXX | TTCAGA |
| Pageland, South Carolina | (34.792517,  -80.415333) | SC-7 | 4.8 | D0U2VACXX | GGAAGA |
| Crossville, Tennessee | (36.119433,  -85.0589) | TN-12 | 6.0 | D0U2VACXX | ACGACTAC |
|  |  | TN-6 | 11.7 | D0U2VACXX | GAATTCA |
|  |  | TN-8 | 3.8 | D0U2VACXX | GGACCTA |
| Summit Lake, Wisconsin | (45.415167,  -89.190017) | WI-6 | 11.8 | D0U2VACXX | GTCAA |
|  |  |  |  |  |  |
| Crossdale, Queensland, Australia | (-27.148033, 152.569135) | AUA-10 |  | C1B5JACXX | CCGGATAT |
| Sarabah, Queensland, Australia | (-28.100028, 153.116677) | AUB-9 |  | C1B5JACXX | TAATA |
| Brays Creek, New South Wales, Australia | (-28.416469, 153.200021) | AUC-4 |  | C1B5JACXX | TTCCTGGA |
| *Not applicable* | | C1B5JACXX  LibraryBlank |  | C1B5JACXX | CCAGCT |
| Mont-Sur-Rolle, Switzerland |  | CHR-3 |  | C1B5JACXX | AAAAGTT |
|  |  | CHR-4 |  | C1B5JACXX | TTCAGA |
| *Not applicable* | | D0U2VACXX  LibraryBlank |  | D0U2VACXX | TAGCATGC |
| Homestead, Florida | (25.541789,  -80.412844) | FLA-1 |  | C1B5JACXX | CGCTT |
|  |  | FLA-3 |  | C1B5JACXX | GAATTCA |
|  |  | FLA-4 |  | C1B5JACXX | TGGTACGT |
|  |  | FLA-5 |  | C1B5JACXX | GGAAC |
|  |  | FLA-6 |  | C1B5JACXX | GGAAGA |
|  |  | FLA-7 |  | C1B5JACXX | GCGGAAT |
|  |  | FLA-8 |  | C1B5JACXX | GGATTGGT |
| Fort Pierce, Florida | (27.374365,  -80.416042) | FLD-3 |  | C1B5JACXX | TGCAAGGA |
|  |  | FLD-4 |  | C1B5JACXX | GAGGA |
|  |  | FLD-5 |  | C1B5JACXX | GCCAGT |
|  |  | FLD-6 |  | C1B5JACXX | CTACGGA |
| Arcadia, Florida | (27.085217,  -81.794967) | FLG-10 |  | D0U2VACXX | CTATTA |
|  |  | FLG-7 |  | D0U2VACXX | ATCGTA |
|  |  | FLG-8 |  | D0U2VACXX | CATCGT |
|  |  | FLG-9 |  | D0U2VACXX | CGCGGT |
| Budapest, Hungary |  | HUA-7 |  | C1B5JACXX | GAACTTC |
| Cegléd, Hungary |  | HUB-7 |  | C1B5JACXX | GTACTT |
| Nyíregyháza, Hungary |  | HUC-7 |  | C1B5JACXX | GCTCTA |
| Cambridge, Iowa | (41.874983, -93.482783) | IA-10 |  | D0U2VACXX | GGAAC |
|  |  | IA-7 |  | D0U2VACXX | CATCT |
|  |  | IA-8 |  | D0U2VACXX | CCTAC |
| Greenup, Illinois | (39.23775,  -88.151217) | IL-10 |  | D0U2VACXX | CTAGC |
|  |  | IL-11 |  | D0U2VACXX | ACAAA |
|  |  | IL-12 |  | D0U2VACXX | TTCTC |
|  |  | IL-6 |  | D0U2VACXX | TGCGA |
|  |  | IL-7 |  | D0U2VACXX | CGAT |
|  |  | IL-8 |  | D0U2VACXX | CGCTT |
|  |  | IL-9 |  | D0U2VACXX | TCACC |
| Holden, Louisiana | (30.468783,  -90.6429) | LA-11 |  | D0U2VACXX | TCGAAGA |
|  |  | LA-15 |  | D0U2VACXX | ATTGA |
|  |  | LA-6 |  | D0U2VACXX | CGCTGAT |
|  |  | LA-8 |  | D0U2VACXX | CTACGGA |
| Clinton, Maine | (44.632296,  -69.507862) | MEB-1 |  | C1B5JACXX | CAGA |
|  |  | MEB-2 |  | C1B5JACXX | CTGTA |
|  |  | MEB-3 |  | C1B5JACXX | ATGCCT |
|  |  | MEB-4 |  | C1B5JACXX | GGTGT |
|  |  | MEB-5 |  | C1B5JACXX | AGGAT |
|  |  | MEB-6 |  | C1B5JACXX | ATCGTA |
|  |  | MEB-7 |  | C1B5JACXX | ATTGGAT |
|  |  | MEB-8 |  | C1B5JACXX | ACGTGGTA |
| Lisbon, Maine | (44.013321,  -70.069435) | MEC-2 |  | C1B5JACXX | GTATT |
|  |  | MEC-4 |  | C1B5JACXX | ACGACTAC |
|  |  | MEC-5 |  | C1B5JACXX | TCAC |
|  |  | MEC-8 |  | C1B5JACXX | ACAGGGAA |
| Calais, Maine | (45.157841,  -67.289035) | MEE-1 |  | C1B5JACXX | AACT |
|  |  | MEE-2 |  | C1B5JACXX | ACCGT |
|  |  | MEE-3 |  | C1B5JACXX | TATTTTT |
|  |  | MEE-4 |  | C1B5JACXX | TAGCATGC |
|  |  | MEE-5 |  | C1B5JACXX | ATTGA |
|  |  | MEE-6 |  | C1B5JACXX | CATCGT |
|  |  | MEE-7 |  | C1B5JACXX | CATAAGT |
|  |  | MEE-8 |  | C1B5JACXX | CCATGGGT |
| Pickford, Michigan | (46.172383,  -84.36515) | MIA-10 |  | D0U2VACXX | CTTGCTT |
|  |  | MIA-11 |  | D0U2VACXX | ATGAAAC |
|  |  | MIA-12 |  | D0U2VACXX | AAAAGTT |
|  |  | MIA-7 |  | D0U2VACXX | GAGATA |
|  |  | MIA-9 |  | D0U2VACXX | TATTTTT |
| Ashley, Michigan | (43.153767,  -84.503683) | MIB-10 |  | D0U2VACXX | CATAAGT |
| Rice, Minnesota | (45.793567,  -94.24465) | MN-10 |  | D0U2VACXX | AGGAT |
|  |  | MN-7 |  | D0U2VACXX | AGGC |
|  |  | MN-9 |  | D0U2VACXX | TCAC |
| Odessa, Missouri | (38.968433,  -93.879417) | MOA-11 |  | D0U2VACXX | TTCCTGGA |
|  |  | MOA-6 |  | D0U2VACXX | CGTGTGGT |
|  |  | MOA-7 |  | D0U2VACXX | GCTGTGGA |
|  |  | MOA-8 |  | D0U2VACXX | GGATTGGT |
| St. Louis, Missouri | (38.386347, -90.25932) | MOB-3 |  | C1B5JACXX | ATGAAAC |
|  |  | MOB-6 |  | C1B5JACXX | CTATTA |
|  |  | MOB-7 |  | C1B5JACXX | CGGTAGA |
| Hannibal, Missouri | (39.709343,  -91.500199) | MOC-1 |  | C1B5JACXX | GCGT |
|  |  | MOC-2 |  | C1B5JACXX | GTAA |
|  |  | MOC-4 |  | C1B5JACXX | AGTGGA |
|  |  | MOC-5 |  | C1B5JACXX | CATCT |
|  |  | MOC-8 |  | C1B5JACXX | CGCGGAGA |
| Chesterfield, New Hampshire | (42.890685,  -72.527665) | NHA-2 |  | C1B5JACXX | CCACAA |
|  |  | NHA-3 |  | C1B5JACXX | GTCGATT |
|  |  | NHA-4 |  | C1B5JACXX | CGCCTTAT |
|  |  | NHA-5 |  | C1B5JACXX | TACAT |
|  |  | NHA-6 |  | C1B5JACXX | TAACGA |
| Washington, New Jersey | (40.740027,  -74.953111) | NJA-1 |  | C1B5JACXX | TGCA |
|  |  | NJA-2 |  | C1B5JACXX | AGCCC |
|  |  | NJA-3 |  | C1B5JACXX | CTTCCA |
|  |  | NJA-4 |  | C1B5JACXX | AATATGC |
|  |  | NJA-5 |  | C1B5JACXX | GATC |
|  |  | NJA-6 |  | C1B5JACXX | ACCTAA |
|  |  | NJA-7 |  | C1B5JACXX | ACGTGTT |
|  |  | NJA-8 |  | C1B5JACXX | AACCGAGA |
| Middle Township, New Jersey | (39.149171,  -74.851037) | NJD-1 |  | C1B5JACXX | CTCC |
|  |  | NJD-2 |  | C1B5JACXX | TTCTC |
|  |  | NJD-3 |  | C1B5JACXX | GCTTA |
|  |  | NJD-4 |  | C1B5JACXX | AACGCCT |
| Freehold, New Jersey | (40.226333,  -74.257833) | NJF-10 |  | D0U2VACXX | CAGA |
|  |  | NJF-7 |  | D0U2VACXX | CTCC |
|  |  | NJF-8 |  | D0U2VACXX | TGCA |
| Wallkill, New York | (41.629,  -74.18235) | NJI-7 |  | D0U2VACXX | AGCCC |
| Lawrencetown, Nova Scotia, Canada | (44.908797,  -65.177264) | NSA-1 |  | C1B5JACXX | CTAGC |
|  |  | NSA-3 |  | C1B5JACXX | GGACCTA |
|  |  | NSA-6 |  | C1B5JACXX | GTTGAA |
|  |  | NSA-7 |  | C1B5JACXX | TCGAAGA |
|  |  | NSA-8 |  | C1B5JACXX | TATCGGGA |
| Lower Sackville, Nova Scotia | (44.754723,  -63.671654) | NSB-1 |  | C1B5JACXX | TCACC |
|  |  | NSB-2 |  | C1B5JACXX | TAGGAA |
|  |  | NSB-5 |  | C1B5JACXX | GTCAA |
|  |  | NSB-6 |  | C1B5JACXX | TAGCGGA |
|  |  | NSB-7 |  | C1B5JACXX | GTGAGGGT |
| Oak Hill, Ohio | (38.909567,  -82.728417) | OHB-10 |  | D0U2VACXX | CGCCTTAT |
|  |  | OHB-7 |  | D0U2VACXX | TGGTACGT |
|  |  | OHB-8 |  | D0U2VACXX | TCTCAGTC |
|  |  | OHB-9 |  | D0U2VACXX | CCGGATAT |
| Connellsville, Pennsylvania | (40.0785,  -79.61485) | PAA-7 |  | D0U2VACXX | GCTCTA |
|  |  | PAA-8 |  | D0U2VACXX | CCACAA |
|  |  | PAA-9 |  | D0U2VACXX | GCTTA |
| Fall River, Massachusetts | (41.683117,  -71.134283) | RIA-10 |  | D0U2VACXX | TAGGAA |
|  |  | RIA-6 |  | D0U2VACXX | GTAA |
| Pageland, South Carolina | (34.792517,  -80.415333) | SC-10 |  | D0U2VACXX | TAACGA |
|  |  | SC-6 |  | D0U2VACXX | GCCAGT |
|  |  | SC-8 |  | D0U2VACXX | GTACTT |
|  |  | SC-9 |  | D0U2VACXX | GTTGAA |
| Crossville, Tennessee | (36.119433,  -85.0589) | TN-10 |  | D0U2VACXX | AACGCCT |
|  |  | TN-11 |  | D0U2VACXX | AATATGC |
|  |  | TN-13 |  | D0U2VACXX | TCGTT |
|  |  | TN-7 |  | D0U2VACXX | GAACTTC |
|  |  | TN-9 |  | D0U2VACXX | GTCGATT |
| Summit Lake, Wisconsin | (45.415167,  -89.190017) | WI-10 |  | D0U2VACXX | ACCTAA |
|  |  | WI-7 |  | D0U2VACXX | TAATA |
|  |  | WI-8 |  | D0U2VACXX | TACAT |

**Supplementary Table 2**. Pairwise F_ST_ estimated between three genetic clusters defined by principal components analysis. All values were significant (*P* < 0.01).

|  | Southeastern | Western | Northeastern |
| --- | --- | --- | --- |
| Southeastern | - |  |  |
| Western | 0.0197 | - |  |
| Northeastern | 0.0152 | 0.0103 | - |

**Supplementary Table 3**. Pairwise F_ST_ estimated between genetic clusters defined by ADMIXTURE analysis with *K* = 3. All values were significant (*P* < 0.01).

|  | Southeastern | Western | Northeastern |
| --- | --- | --- | --- |
| Southeastern | - |  |  |
| Western | 0.0189 | - |  |
| Northeastern | 0.0147 | 0.0117 | - |

**Supplementary Table 4**. Pairwise F_ST_ estimated between genetic clusters defined by ADMIXTURE analysis with *K* = 4. All values were significant (*P* < 0.01).

|  | Southeastern | Southwestern | Northeastern | Midwestern |
| --- | --- | --- | --- | --- |
| Southeastern | - |  |  |  |
| Southwestern | 0.0177 | - |  |  |
| Northeastern | 0.0151 | 0.0116 | - |  |
| Midwestern | 0.0356 | 0.0225 | 0.0270 | - |
